# Intelligent analysis of the quality of education through teaching practices on virtual campuses

**DOI:** 10.1007/s10212-022-00649-2

**Published:** 2022-11-10

**Authors:** Lucia Alvarez-Blanco, Adrian Castro-Lopez, Antonio Cervero

**Affiliations:** 1 Department of Educational Sciences, University of Oviedo, Oviedo, Spain; 2 Department of Business Administration, University of Oviedo, Oviedo, Spain; 3 Department of Psychology, University of Oviedo, Oviedo, Spain

**Keywords:** Higher education, University, Electronic learning, Blended learning, Fuzzy inference systems

## Abstract

ICTs have been increasingly involved in teaching–learning processes due to the potential offered by the tools as well as to the set of demands derived from the political and health situations of the social environment. In this sense, the introduction of virtual campuses as complex systems that centralize the entire technological component that complements traditional teaching processes has meant a change of paradigm with repercussions at the teaching and pedagogical level. In this context, the purpose of this study aims to analyze students’ perception of the use of virtual campus and how to enhance the quality of the educational process using intelligent systems. For this purpose, 318 students that use virtual campus have been surveyed. The results show that there are three variables that predominantly influence the quality of teaching–learning processes using virtual campuses: frequent contact with teachers through the platform, the digital competence of the student, and the adaptation of training content to the students’ prior knowledge. This information can be useful, as it allows them to establish guidelines to guide the practices of their teaching teams in technological environments, guaranteeing the suitability of the teaching–learning process and improving the evaluation processes and the assessment of their own educational work.

## Introduction

The political, economic, and social changes in Western societies over the mid-twentieth century meant that communities undergoing this kind of transformation were called, according to Bell (1973), post-industrial societies. In this context, Drucker (1976) developed the concept of “knowledge society,” focusing attention on its new characteristics, centered on the following: an expanded educational, technical, and scientific sphere; greater complexity in the processes of information and knowledge circulation; and major labor and business changes, which aim to promote a continuous process of product and service innovation (Saikkonen & Kaarakainen, 2021).

The need for all institutions, both public and private, to adapt to this new type of society entails important organizational changes, but especially relevant in those organizations which, like the University, have the creation and transmission of knowledge at the core of their function. Thus, to practically develop the postulates that favor the knowledge society, university institutions have previously had to undergo a process of internal reorganization. In Europe and Spain in particular, this has been achieved through the development of the European Higher Education Area (EHEA). The establishment this common frame of reference, which affects not only Spanish universities but also universities in the countries that are part of the EHEA, aims to restructure based on five fundamental pillars: comparability of studies, university autonomy, financing, the establishment of quality criteria, and the mobility of those involved.

In fact, the importance of the continuous effort to improve the efficiency in teaching–learning procedures is where pedagogical approaches involving the integration of information and communication technologies (ICTs) play an important role (Akhmedov, 2021), as it changes, among other aspects, the way of communicating, relating to content, and creating and offering information (Bartolomé & Grané, 2013).

As a result of the strong (if not obligatory) commitment to the use of technological tools in training processes, universities have followed the trend and have adopted e-campuses in all their academic programs, functioning as fully integrated packages with all the necessary logistics to develop appropriate technological devices and modules. In all of them, associated with digital competence (Zhaoa et al., 2021), the most widely used at this educational level is Moodle (Silva et al., 2016), which has allowed to develop a real digital higher education concept (Llorens, 2014).

For this reason, part of the evaluation of the pedagogical program using e-campuses should consider which tools are used (Avello et al., 2016) and its purpose, but keeping in mind that the study of the educative development must extend further than the e-campus and its tools, including a more pedagogical aspect that considers how the habitual use of online training processes conditions the teaching planning process itself, the methodology, or the communicative interactions (Torres & Moreno, 2013), all aspects that are already contemplated in university models of global quality assessment (Marciniak & Gairín, 2019; Vega et al., 2017).

Likewise, it is fundamental to know the perspective of the student, without underestimating the perception of the teacher, because both the principles of e-learning and the EHEA fundamentals place special emphasis on placing the learner within the core of the teaching–learning process. Thus, the spotlight shifts from the teaching approach to the student’s learning approach following the principles of constructivist models that make special mention of the acquisition of competencies by the learner at his or her own individualized pace (Herrera, 2015), which in turn leads to significant changes in the lecturer’s role from a knowledge transmitter to a knowledge facilitator (Conole, 2013).

Taking all this into account, this study attempts determine how students perceive their use of e-campuses in higher education to find out which are the main variables that determine the quality of the teaching–learning process. For this purpose, techniques based on fuzzy inference systems (FIS) that handle the uncertainty associated with real-life decision problems by incorporating linguistic terms that are much more similar to human thinking.

## Method

According to population characteristics and the phenomenon analyzed, an ex-post-facto research design has been chosen, in which there is no manipulation of variables but rather selection of values based on an event that has already occurred (Fontes et al., 2010).

### Sample

The sample consisted of 318 subjects, mostly students from on-site universities (89.9%), and the rest (10.1%) from online or distance learning universities, both public and private, on-site, and online. Due to this distribution, there is a greater presence of students from public universities (93.1%) than from private universities (6.9%).

Regarding the sociodemographic profile of the sample, there is a higher proportion of women (70.1%) than men (29.9%), with a median age of 24 years, and the students are mainly undergraduates (88.4%), with the remaining 11.6% studying for a master’s degree. Regarding previous education, approximately half of the students have a high school diploma (49.7%), 14.2% have obtained a vocational training degree, and 36.1% higher education degree (23.6% degree, 11.6% a master’s degree, and 0.9% doctoral degree), being especially noteworthy that 53.8% of students perform some type of remunerated employment outside home. In terms of knowledge area, most students are from the Social and Legal Sciences sector (73%), followed by Health Sciences (13%), Arts and Humanities (6%), Sciences (4%), and Engineering and Architecture (4%), an aspect that responds to the greater number of degrees and students of the first area in this university.

### Instrument

From a positivist paradigm (Creswell, 2014), the survey has been adopted as the priority technique to collect information. Specifically, and associated with this, a questionnaire has been designed ad hoc entitled: “Analysis of higher education students’ perception of e-campuses in the European Higher Education Area (EHEA).” This instrument is made up of nine classification variables and seven dimensions that integrate items according to (1) availability of technological resources at home, (2) teaching planning, (3) contents, (4) methodology, (5) communication, (6) evaluation, and (7) digital competence.

The demographical variables refer to aspects such as sex, age, university where studies are being carried out, ownership of the university, studies being carried out, type of studies (degree or equivalent, master's or doctorate), course, level of studies completed up to now, and remunerated work outside the home.

The dimension of technological resources at home is made up of five dichotomous response items (yes/no) that ask about if the family own a computer at home, whether it is for personal or shared use, availability of a tablet, Internet connection, or cell phone with data connection.

The other dimensions are composed of a diverse range of number of items. However, all of them, teaching planning (4 items), contents (7 items), methodology (5 items), communication (5 items), evaluation (5 items), and digital competence (4 items), are answered according to a four-option Likert-type scale (more appropriate for our objectives than the traditional five-position scale, as it allows dichotomizing the variables) from 1 (strongly disagree) to 4 (strongly agree).

In this sense, teaching planning dimension is concerned with whether pedagogical course descriptors, such as competencies and learning outcomes (Gonçalves et al., 2016), contents (De Jong et al., 2020), methodology, or evaluation criteria, are available on the virtual campus for student consultation (Baldwin & Ching, 2019).

The content dimension (García et al., 2019) tries to determine if the contents are adapted to the students’ previous knowledge, if they use the terminology of the professional discipline, if they are updated, if they are prepared with didactic criteria, if they are available in different formats, including some suitable for printing, and if the student has access to them.

The methodology dimension aims to determine whether the exercises and activities refer to real problems and situations that a professional may encounter, whether they encourage the presentation of ideas and discussion, critical thinking, and reflection (López-Gil & Bernal-Bravo, 2019), and whether the teacher promotes motivation or provides advice and guidance.

The communication dimension assesses the relationship between the student and the teacher (Cabanillas et al., 2018), and seeks information on whether the teacher frequently contacts the student, whether communication is fluid, responses are quick and satisfactory, and whether the teacher requests an evaluation of the course.

The evaluation dimension (Rodríguez-García et al., 2019) studies whether the teacher publishes on campus self-evaluation activities like those of the performance test, whether he/she assigns specific weight to student participation in tutoring or to the completion of online exercises, whether individual or group, or whether he/she provides his/her grades to the student using digital tools.

The digital competence dimension analyzes the student’s perception of his or her own mastery of the use of electronic devices (López-Meneses et al., 2020) and the teacher’s training and attitude in this area, in addition to concluding whether the use of e-campus as an educational tool is a quality criterion.

Finally, a dichotomous item is included that asks whether the student has ever thought of abandoning the university studies in which he/she is enrolled and an open-ended question on comments and suggestions is added, related to the content of the questionnaire or to the student’s view of the use of the virtual campus.

### Procedure

The present study is framed within the quantitative perspective in the field of educational research. For this reason, to ensure accessibility to students, a questionnaire was administered to a group of undergraduate and master’s degree students who had access to the e-campus in most subjects they were taking. Thus, the selection of subjects responds to a non-probabilistic sampling of the “snowball” or “chain sampling” type (Pérez-Luco et al., 2017).

Said questionnaire has been made available to students through two channels: on the one hand, in printed format to be filled out by students in person in the different subjects and on the other hand, published in the online form management application of Google Drive, being disseminated through different social networks (Facebook, Twitter, LinkedIn) with direct request to the official profiles of the universities in these networks.

### Data analysis

The descriptive analysis of the data and the decision trees was performed with the SPSS V. 27 statistical software. The classification trees are a data mining method that allows creating a classification model based on flow diagrams. So, they allow categorizing cases and predicting values of a criterion variable based on values of predictor variables, which explains the behavior with respect to a decision, facilitating the interpretation and the knowledge understanding involved into the multiple-criteria decision-making (MCDM) process (Berlanga et al., 2013). In addition, the measurement instrument has been validated with Cronbach’s Alpha coefficient of 0.949; therefore, this measurement scale is adequate.

Afterwards, supported by Matlab Fuzzy Logic Toolbox™ program, the proposed MCDM model is supported by FIS to assess e-campus quality. For this purpose, this information collected in the decision trees is used as an input base, including this previous knowledge for the realization of the knowledge base included in FIS and with which to design the fuzzy inference maps.

## Results

To carry out the classification tree, the dependent variable taken as the dependent variable was item (ECQUALITY): “In university teaching and learning, the utilization of the e-campus as a support tool enhances the educational quality of the education received,” as it allows to extract a direct assessment of the student regarding the overall teaching–learning process at a global level. Then, to facilitate the analyses, the initial four-point Likert-type response scale was dichotomized, with values 1 and 2 being understood as a negative evaluation of the use of the virtual campus, while values 3 and 4 implied a positive evaluation. Thus, two different profiles were obtained according to their degree of satisfaction (or dissatisfaction) with the teaching use of the virtual campus.

**Fig. 1:**
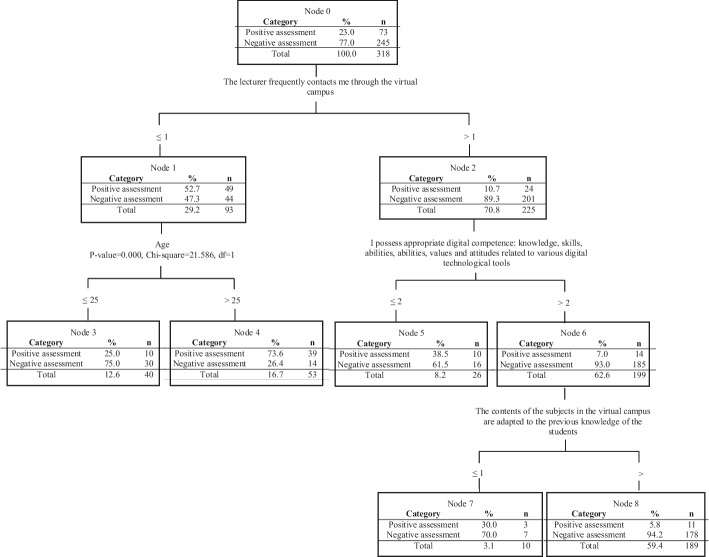
Decision tree

This allowed us to validate a model that determines which variables give the highest quality to the educational process implemented through the virtual campus, with a 94.3% success rate in the assignment. According to Fig. 1, the most relevant variables that explain the phenomenon are arranged in different hierarchical levels.

The classification tree shows that the item with the highest predictive capacity in terms of the positive evaluation as a quality element of the e-campus is the contact frequency between the teacher and the student through the virtual campus (χ^2^ = 65.697; *p* < 0.001). Thus, if the evaluation of the frequency of contact with the teacher (FREQ1) is greater than 1, 89.3% of the subjects are classified in the situation of positive evaluation of the campus, a percentage that drops to 47.3% among those who express total disagreement with the variable that indicates that the teacher frequently contacts them.

Nonetheless, at a second level, age seems to function as a modulating element (χ^2^ = 21.586; *p* < 0.001), since as can be seen among those who rate the frequency of teacher contact very negatively, the fact of being under 25 years of age allows them to maintain a positive consideration of the use of the campus as a teaching–learning tool (73.6%), which does not occur among students over 25 years of age in whose group the positive evaluation drops significantly (26.4%).

A similar effect at the same level, but among students who value more positively the frequency of teaching contact (> 1), can be observed in the variable associated with the self-perceived level concerning digital skills competence (TECH3), defined on the basis of the knowledge and skills, and abilities, related to digital technological instruments (χ^2^ = 23.834; *p* < 0.001). Thus, those that assess their own technological competence considered adequate (> 2) positively value the e-campus (93%) if it is combined with frequent contact, a fact that does not occur with the same intensity among students who rate their technological competence as deficient (61.5%).

Finally, at a third level, the adjustment of the contents to the students’ prior knowledge becomes relevant. Thus, if the variable “the program course materials on virtual campus are adapted to the students” background knowledge (CONTK1) is not valued excessively negatively (> 1) (χ^2^ = 8.490; p = 0.021), a successful assessment of the e-campus as an important quality component will be obtained. (94.2%), with a satisfactory evaluation (70%) decreasing if this adaptation does not occur.

**Fig. 2:**
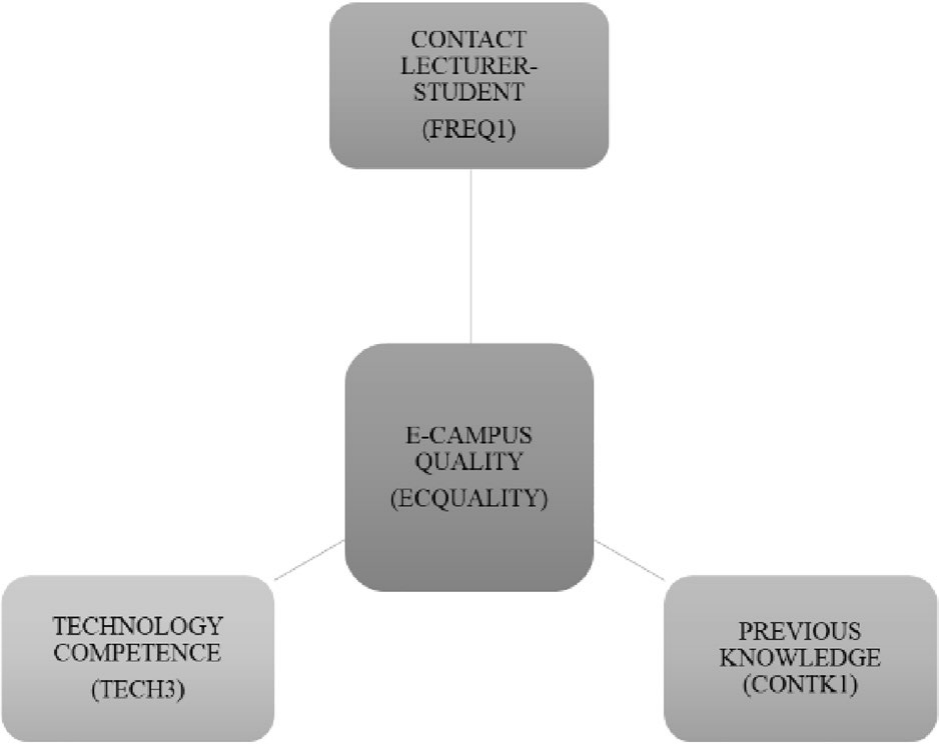
Proposed research model

As a result of these analyses and the design of the decision tree itself, it has been possible to establish the illustrated model that can be seen in Fig. 2, relating to the quality assessment on virtual educational process.

### Fuzzy inference systems

Nowadays, students and academics are increasingly involved in making multi-criteria decisions, mostly hardly quantifiable due to their high degree of subjectivity in determining the importance of each of the variables that affect the final valuation (Guarini et al., 2021). Therefore, to address MCDM problems, fuzzy techniques are becoming commonplace, through the development of a fuzzy language that is closer to people’s thinking mode (Castro-López et al., 2021). According to Lazzari and Moulia (2012), reality is fraught with vagueness and uncertainty, so that people’s actions and behaviors can hardly be described with accuracy and precision. Therefore, reality cannot be studied in absolute conditions through probabilistic approaches that attempt to constrain multi-criteria decision problems to a rigid and static mathematical model, thus missing out on valuable information. Techniques based on fuzzy systems are aimed at dealing adequately with the imprecision and uncertainty of system identification and modelling (Yannis et al., 2020) thus allowing for a better evaluation of the results.

Therefore, the so-called fuzzy inference systems (FIS), introduced by Zadeh (1965), make it possible that the uncertainty concept can be added to the multi-criteria evaluation framework, achieving a better, easier, and interpretable approach (Alonso-Moral et al., 2021). In such systems, these linguistic variables enable the treatment of information, both quantitative and qualitative, because the labels chosen acquire values associated with the ordinary language (Driankov et al., 2013; Villa-Silva et al., 2021). In the particular case of the evaluation of virtual educational environments, the subjectivity associated with certain variables may be high. For this reason, treating the variables linguistically makes it possible enhancing the handling of the assessment criteria provided by the students. Furthermore, it can be shown how fuzzy design in this domain is conducive to a more accurate interpretation as well as more consistent evaluation behavior compared to simpler evaluation systems. This will require defining the FIS knowledge database on the basis of the know-how of the experts. In addition, the experts will mutually decide what rank and type of fuzzy labels should be used to divide the domains in each variable of the model. They will then discuss the rules to be constructed from those fuzzy variables, able to describe as much as possible the proposed framework. This method requires the following steps to be carried out: (1) domain partitioning and knowledge gathering for the design of the evaluation model rule base; (2) partitioning of the variable’s domain into the inference subsystems in the assessment model; and (3) extraction of the expert knowledge to define the rule base in the model.

To this end, a panel of experts made up of 5 specialists in the sector was created to help solve the problem by partitioning the variables and defining the rule base. First, to standardize the method, it has been decided to set the equal number of input and output labels (trapezoidal fuzzy numbers) to assess the e-campus quality model. To achieve a consensus between experts, pairwise scales (highly disapprove, partly disapprove, partly approve, highly agree) were used to define the labels. Then, considering the arithmetic mean, the experts decided to use the following labels for the input variables (bad (B), average (A), good (G)) and the following labels for the output variable (very bad (VB), bad (B), average (A), good (G), very good (VG)).

Then, the semantic representation labels were assigned to trapezoidal fuzzy numbers, because they are robust enough to address the imprecise nature of language assessments (Mencar & Fanelli, 2008). Furthermore, it was considered to use robust fuzzy partitions, since they are the optimal choice regarding understandability due to the fact that they fulfill important semantic restrictions such as differentiability, standardization, overlapping, and coverage. Then, partitioning the variable domain into the fuzzy model, it is possible to use a linguistic representation model based on 2-tuples that enhances its systematic approach through fuzzy numbers (Deng et al., 2020; Wang & Wang, 2020). In this case, the linguistic data can be represented and handled by a pair value (si, αi), whereas “si” refers to a linguistic expression and “i” is a numeric score assessed on the range (− 0.5, 0.5) which represents the symbolic translation (Fernández et al., 2010). According to a previously defined scale preference, a group of specialists will reach an agreement on the preferences to be given to each possible label that could be allocated in each variable within the proposed model (Castro-López et al., 2021).

Afterwards, based on the structure agreed upon by the specialists, the semantic for each label is developed, considering the criteria defined and thus ensuring a significant partitioning of each variable. Table 1 shows the final partitioning for the variables that have an influence on the evaluation for virtual campuses.

**Table 1:** Labels definition with trapezoidal fuzzy numbers

Domain partitioning variable	Label	Trapezoidal fuzzy numbers
Input	Bad (B)	(0.0 0.0 0.4 0.5)
	Average (A)	(0.4 0.5 0.7 0.8)
	Good (G)	(0.7 10)
Output	Very bad (VB)	(0 1 2 3)
	Bad (B)	(2 3 4 5)
	Average (A)	(4 5 6 7)
	Good (G)	(6 7 8 9)
	Very good (VG)	(8 10)

Finally, an extension-based elicitation method (Dubois & Prade, 1980) was used to extract the expert knowledge for defining the rule bases in the inference subsystems within this approach to the assessment model. At the first stage, experts have been requested to select their preferred output labels in accordance with the partitioning defined above for the FIS of the suggested model for each input label combination. Table 2 lists the ratings given to the input variables in the fuzzy model provided by the experts.

**Table 2:** Linguistic assessments given for the fuzzy model

**FREQ1**	**CONTK1**	**TECH3**	Exp1	Exp2	Exp3	Exp4	Exp5	Expert 1				Expert 2				Expert 3				Expert 4				Expert 5			
B	B	B	VB	VB	VB	B	VB	0	0	2	3	0	0	2	3	0	0	2	3	2	3	4	5	0	0	2	3
		A	VB	B	VB	B	B	0	0	2	3	2	3	4	5	0	0	2	3	2	3	4	5	2	3	4	5
		G	VB	B	VB	B	A	0	0	2	3	2	3	4	5	0	0	2	3	2	3	4	5	4	5	6	7
	A	B	VB	VB	VB	A	B	0	0	2	3	0	0	2	3	0	0	2	3	4	5	6	7	2	3	4	5
		A	B	B	VB	A	A	2	3	4	5	2	3	4	5	0	0	2	3	4	5	6	7	4	5	6	7
		G	B	A	VB	G	A	2	3	4	5	4	5	6	7	0	0	2	3	6	7	8	9	4	5	6	7
	G	B	B	VB	VB	A	B	2	3	4	5	0	0	2	3	0	0	2	3	4	5	6	7	2	3	4	5
		A	B	B	VB	G	A	2	3	4	5	2	3	4	5	0	0	2	3	6	7	8	9	4	5	6	7
		G	B	A	VB	G	G	2	3	4	5	4	5	6	7	0	0	2	3	6	7	8	9	6	7	8	9
A	B	B	B	B	VB	B	VB	2	3	4	5	2	3	4	5	0	0	2	3	2	3	4	5	0	0	2	3
		A	B	A	B	B	A	2	3	4	5	4	5	6	7	2	3	4	5	2	3	4	5	4	5	6	7
		G	B	A	B	B	B	2	3	4	5	4	5	6	7	2	3	4	5	2	3	4	5	2	3	4	5
	A	B	B	A	B	A	B	2	3	4	5	4	5	6	7	2	3	4	5	4	5	6	7	2	3	4	5
		A	A	A	A	A	B	4	5	6	7	4	5	6	7	4	5	6	7	4	5	6	7	2	3	4	5
		G	A	G	A	G	A	4	5	6	7	6	7	8	9	4	5	6	7	6	7	8	9	4	5	6	7
	G	B	A	A	A	G	A	4	5	6	7	4	5	6	7	4	5	6	7	6	7	8	9	4	5	6	7
		A	G	G	A	G	A	6	7	8	9	6	7	8	9	4	5	6	7	6	7	8	9	4	5	6	7
		G	G	VG	G	VG	G	6	7	8	9	8	9	10	10	6	7	8	9	8	9	10	10	6	7	8	9
G	B	B	B	B	B	VB	B	2	3	4	5	2	3	4	5	2	3	4	5	0	0	2	3	2	3	4	5
		A	B	B	A	B	A	2	3	4	5	2	3	4	5	4	5	6	7	2	3	4	5	4	5	6	7
		G	A	A	A	B	A	4	5	6	7	4	5	6	7	4	5	6	7	2	3	4	5	4	5	6	7
	A	B	B	A	B	A	A	2	3	4	5	4	5	6	7	2	3	4	5	4	5	6	7	4	5	6	7
		A	A	G	A	A	G	4	5	6	7	6	7	8	9	4	5	6	7	4	5	6	7	6	7	8	9
		G	G	G	G	G	G	6	7	8	9	6	7	8	9	6	7	8	9	6	7	8	9	6	7	8	9
	G	B	A	A	G	A	A	4	5	6	7	4	5	6	7	6	7	8	9	4	5	6	7	4	5	6	7
		A	G	G	VG	G	VG	6	7	8	9	6	7	8	9	8	9	10	10	6	7	8	9	8	9	10	10
		G	VG	VG	VG	VG	VG	8	9	10	10	8	9	10	10	8	9	10	10	8	9	10	10	8	9	10	10

From these ratings, applying the arithmetic aggregation approach, according to the extension principle, the “collective preference vectors” associated with the input label combination for this proposed model are reflected in Table 3.

**Table 3:** Collective preference vectors

FREQ1	CONTK1	TECH3	Collective preference vectors			
B	B	B	0.40	0.60	2.40	3.40
		A	1.20	1.80	3.20	4.20
		G	1.60	2.20	3.60	4.60
	A	B	1.20	1.60	3.20	4.20
		A	2.40	3.20	4.40	5.40
		G	3.20	4.00	5.20	6.20
	G	B	1.60	2.20	3.60	4.60
		A	2.80	3.60	4.80	5.80
		G	3.60	4.40	5.60	6.60
A	B	B	1.20	1.80	3.20	4.20
		A	2.80	3.80	4.80	5.80
		G	2.40	3.40	4.40	5.40
	A	B	2.80	3.80	4.80	5.80
		A	3.60	4.60	5.60	6.60
		G	4.80	5.80	6.80	7.80
	G	B	4.40	5.40	6.40	7.40
		A	5.20	6.20	7.20	8.20
		G	6.80	7.80	8.80	9.40
G	B	B	1.60	2.40	3.60	4.60
		A	2.80	3.80	4.80	5.80
		G	3.60	4.60	5.60	6.60
	A	B	3.20	4.20	5.20	6.20
		A	4.80	5.80	6.80	7.80
		G	6.00	7.00	8.00	9.00
	G	B	4.40	5.40	6.40	7.40
		A	6.80	7.80	8.80	9.40
		G	8.00	9.00	10.00	10.00

To adjust the preference vectors for each of the labels that make up the knowledge database, the distance of each vector to the five labels that make up the output variable has been estimated, according to the following distance formula:

1 $$D\left(\left[{a}_{i},{b}_{i},{c}_{i},{d}_{i}\right]-\left[{a}^{j},{b}^{j},{c}^{j},{d}^{j}\right]\right)=\sqrt{{P}_{a}{\left({a}_{i}-{a}^{j}\right)}^{2}+{P}_{b}{\left({b}_{i}-{b}^{j}\right)}^{2}+{P}_{c}{\left({c}_{i}-{c}^{j}\right)}^{2}+{P}_{d}{\left({d}_{i}-{d}^{j}\right)}^{2}}$$

Here, *P*_a_, *P*_b_, *P*_c_, and *P*_d_ are the weights assigned to fuzzy number estimation; $\left[{\mathrm{a}}_{\mathrm{i}},{\mathrm{b}}_{\mathrm{i}},{\mathrm{c}}_{\mathrm{i}},{\mathrm{d}}_{\mathrm{i}}\right]$ means ith preference vector $\left[{\mathrm{a}}^{\mathrm{j}},{\mathrm{b}}^{\mathrm{j}},{\mathrm{c}}^{\mathrm{j}},{\mathrm{d}}^{\mathrm{j}}\right]$, and jth label represents the initial partition. Furthermore, for the nine collective preference vectors in the table above, the five labels of the output variable partition (very bad (VB), bad (B), average (A), good (G), and very good (VG)), and taking Pa = Pd = 0.15 and Pb = Pc = 0.35, the distances obtained are indicated in Table 4, which have made it possible to identify the output label to be assigned for every one of the 27 possible combinations of answers according to the model proposed for the e-campus quality assessment. Table 4 shows label definition for the knowledge database.

**Table 4:** Label definition for the knowledge database

			Distances					Output
FREQ1	CONTK1	TECH3	D(VB)	D(B)	D(I)	D(G)	D(VG)	labels
B	B	B	0.52	1.75	3.57	5.40	7.25	**VB**
		A	1.43	0.84	2.66	4.50	6.36	**B**
		G	1.79	0.49	2.29	4.13	5.99	**B**
	A	B	1.34	0.94	2.75	4.58	6.44	**B**
		A	2.61	0.39	1.47	3.32	5.17	**B**
		G	3.35	1.12	0.74	2.58	4.44	**I**
	G	B	1.79	0.49	2.29	4.13	5.99	**B**
		A	2.98	0.75	1.11	2.95	4.81	**B**
		G	3.71	1.49	0.38	2.21	4.07	**I**
A	B	B	1.43	0.84	2.66	4.50	6.36	**B**
		A	3.06	0.81	1.02	2.87	4.73	**B**
		G	2.70	0.44	1.39	3.24	5.10	**B**
	A	B	3.06	0.81	1.02	2.87	4.73	**B**
		A	3.80	1.55	0.28	2.13	3.99	**I**
		G	4.90	2.66	0.81	1.02	2.89	**I**
	G	B	4.53	2.29	0.44	1.39	3.26	**I**
		A	5.27	3.03	1.18	0.65	2.53	**G**
		G	6.74	4.50	2.65	0.78	1.06	**G**
G	B	B	1.88	0.38	2.21	4.05	5.91	**B**
		A	3.06	0.81	1.02	2.87	4.73	**B**
		G	3.80	1.55	0.28	2.13	3.99	**I**
	A	B	3.43	1.18	0.65	2.50	4.36	**I**
		A	4.90	2.66	0.81	1.02	2.89	**I**
		G	6.00	3.77	1.92	0.00	1.80	**G**
	G	B	4.53	2.29	0.44	1.39	3.26	**I**
		A	6.74	4.50	2.65	0.78	1.06	**G**
		G	7.84	5.60	3.75	1.88	0.00	**VG**

**Fig. 3:**
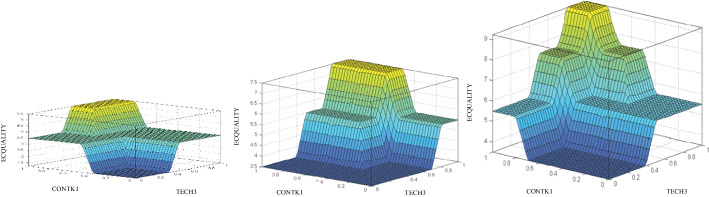
Maps of ECQUALITY assessment results with bad (left), average (center), and good (right) FREQ1

Once the rule base has been defined, the rules are introduced into the FIS through Matlab Fuzzy Logic Toolbox™ which allows to infer the evaluation of the e-campus quality based on the crisp values assigned to its input variables. Furthermore, analyzing the consistency in the assessments obtained by means of the inference charts provided for this model is also simple and intuitive. In those charts, the values are given by the area altitude in every spot. The results achieved can be analyzed by performing the assessment as a function of 2 input variables relative to a fixed value corresponding to the other variable not shown in the chart. Thus, the virtual campus quality assessment can be seen in terms of the digital competence variable (TECH3) and the content variable (CONTK1) according to the different values of the frequency of teaching contact with the student (FREQ1). Thus, in Fig. 3, it can be seen that values of content (CONTK1) or digital competence (TECH3) lower than 0.4 result in a null output, regardless of the assessment in the rest of the variables. However, just as the values for the variables increase, the students’ satisfaction with the e-campus also increases, thus increasing the perception of e-campus quality (ECQUALITY). Such result can be understood as a logical process if it is thought that the interaction with the contents, based on the mastery of digital resources and the high quality and adequate educational design of the materials themselves, can make up to a certain degree for the lack of communication with the lecturer. It can also be seen how, for average and good values of the variable of frequency of contact between the lecturer and the student, good output values are reached in a greater area, which translates into better student evaluations of the quality of the campus. In short, what can be appreciated is the concordance between variables, so that the communicative, didactic, and methodological aspects of the teaching–learning process enhance each other, increasing the overall quality of the educational process.

**Fig. 4:**
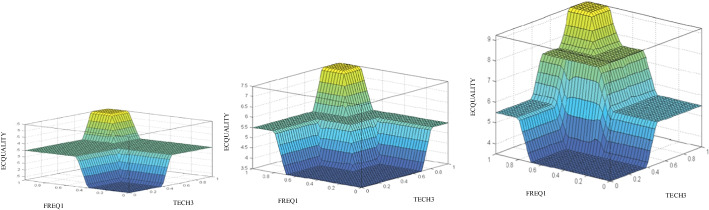
Maps of ECQUALITY assessment results with bad (left), average (center), and good (right) CONTK1

In a second graphical plot, the assessment of the quality of the virtual campus can also be analyzed by taking into consideration the frequency of contact between the lecturer and the student (FREQ1) and the digital competence (TECH3), according to the different values in the content variable (CONTK1) (see Fig. 4). It is observed in this case how values lower than 5 points in the contents variable (CONTK1) only allow obtaining a maximum score of 5.5 points with respect to quality, while if the contents improve, the student’s quality perception of the virtual campus can increase up to 7.5 or 9.5 points for average and good content values, respectively.

**Fig. 5:**
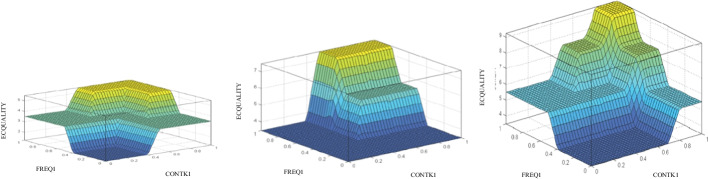
Maps of ECQUALITY assessment results with bad (left), average (center), and good (right) TECH3

Finally, following the logical sequence, Fig. 5 can be obtained, which shows the virtual campus quality assessment solution map showing the contact frequency of the lecturer with the student (FREQ1) and the contents (CONTK1), as a function of the values of the digital competence variable (TECH3).

In this case, the perception of average values in the evaluation of digital competence (TECH3), with scores above 0.4 points, improved results in the assessment of e-campus quality. It is noted that as digital competence scores increase, they increase from 5.5 to 7.5 or to 9.5 points to bad, medium, and good ratings, correspondingly. The reason why students give greater importance to the virtual campus is because they perceive that they are more technologically adept, and this aspect positively influences their relationship with the rest of the variables within the model. Therefore, the e-campus quality assessment will be null whatever the outcome of the digital competency assessment, which shows that at least some minimum adjustments in teaching practice will be necessary for the model to guarantee the quality within the teaching–learning process.

## Discussion and conclusions

The analysis of online teaching–learning processes that has been applied aims to configure an evaluation with a holistic approach (Van Dijk, 2020) that determines which are the main pedagogical and teaching variables that give quality to the educational process in virtual campuses. As can be seen in the model derived from the classification tree, three fundamental variables are identified according to the students’ perspective: the frequent contact of the teacher with the student through the virtual campus (FREQ1), within the communicative dimension; the possession of adequate digital competence by the student, understood defined on the basis of abilities, knowledge, competences, and attitudes related to various digital devices (TECH3), within the dimension of digital competence and referring to the students’ previous training; and the adaptation of the training contents to the students’ previous knowledge (CONTK1), within the dimension of contents of a didactic nature.

The results obtained contextualize, support, and extend some didactic principles and teaching practices widely accepted in the new technological environment of the knowledge society based on different studies (Chang et al., 2007; Instefjord & Munthe, 2017; Borawska-Kalbarczyk et al., 2019; Maasen et al., 2020). Firstly, the frequency of lecturing contact and even the good teacher-student relationship has been pointed out recurrently in the academic literature. García-Valcárcel (2008) in a study involving lecturers’ comments on the importance of the use of technological tools for communicative purposes, such as forums or e-mail, for the improvement of tutorials and to determine, according to Bernardo et al. (2016) determine how a fluid and satisfactory relationship with lecturers reduces the probability of university dropout.

Furthermore, it seems obvious that students’ previous background courses using ICTs should lead to a greater appreciation of ICTs as a teaching methodology. In this sense, the opinion is shared by Centeno and Cubo (2013), which identify a significant relationship among digital competence, ICT awareness, and the e-learning framework, which have led many universities to integrate the development of digital competence in their educational programs (Roig & La-Neve, 2011).

The results of the current study are intended to complement those of the previous study by Cervero et al. (2020), which uses a similar methodology but has a substantial difference in terms of the assessment of the quality of the campus by the selected sample. Thus, in the previous study, the majority of students rated positively the e-campus quality (82.6%), while in the current study, most of the students surveyed rated the quality of the campus negatively (77%). Thus, the new analysis originated by the classification tree includes the evaluation of the elements that give quality to the campus, but from a perspective in which the majority believe that it is deficient, completing the global analysis of quality and giving greater weight to the didactic and pedagogical variables cited by the most demanding students or those with the most difficulties.

Finally, it is worth mentioning the idea that new knowledge should be based on previous knowledge, corroborating the results obtained with this basic pedagogical principle, which is widely accepted and underlies solidly established concepts like Bruner’s scaffolding metaphor or Vygotsky’s zone of proximal development (ZPD). Thus, new contributions focus on how this adaptation can be developed through the contents or materials provided by technological tools (Gordillo & Fernández, 2009), which implies in the educational field a whole process of reformulation of the field of instructional design (Zapata-Ros, 2015).

The practical implications of this study are particularly noteworthy, since it allows institutions to establish guidelines to guide the practices of their teaching teams in technological environments, guaranteeing the suitability of the teaching–learning process. It also provides guidelines to university institutions so that they can design their teacher training plans, focusing on the most relevant aspects of educational practice. Furthermore, it facilitates decision-making to establish and prioritize certain funding channels that, depending on the needs in terms of infrastructure, personnel, or pedagogical renovation, allow for an increase in the educational quality service.

Despite the excellent results obtained in the study, these should be taken with caution considering some aspects that should be strengthened. In this sense, it would be necessary to broaden the sample within the institutions analyzed and extend it to other universities, as well as to balance it according to sex, area of knowledge, and age. Finally, other methods of analysis based on artificial intelligence could be introduced, which would allow us to re-elaborate the model according to the data obtained in the new contexts where the use of ICT in education has increased, if not imposed, according to the latest socio-health requirements.
